# A Giant Left Atrial Myxoma

**DOI:** 10.1155/2014/819052

**Published:** 2014-12-21

**Authors:** Medhat F. Zaher, Sharad Bajaj, Mirette Habib, Emile Doss, Michael Habib, Mahesh Bikkina, Fayez Shamoon, Wissam N. Hoyek

**Affiliations:** ^1^Department of Cardiology, St. Joseph's Regional Medical Center, 703 Main Street, Paterson, NJ 07503, USA; ^2^Department of Cardiology, Staten Island University Hospital, 475 Seaview Avenue, Staten Island, NY 10305, USA

## Abstract

Atrial myxomas are the most common primary cardiac tumors. Patients with left atrial myxomas generally present with mechanical obstruction of blood flow, systemic embolization, and constitutional symptoms. We present a case of an unusually large left atrial myxoma discovered incidentally in a patient with longstanding dyspnea being managed as bronchial asthma.

## 1. Introduction

Myxomas are the most common primary neoplasms of the heart. The clinical presentation varies from asymptomatic incidental masses to serious life-threatening cardiovascular complications. Some cases are difficult to diagnose, as symptoms can be nonspecific. Although the majority of cases are diagnosed by transthoracic echocardiogram, we present a case of an unusually large left atrial myxoma discovered incidentally on an abdominal CT scan in a patient with longstanding exertional dyspnea being managed as bronchial asthma.

## 2. Case Presentation

**Figure 1:**
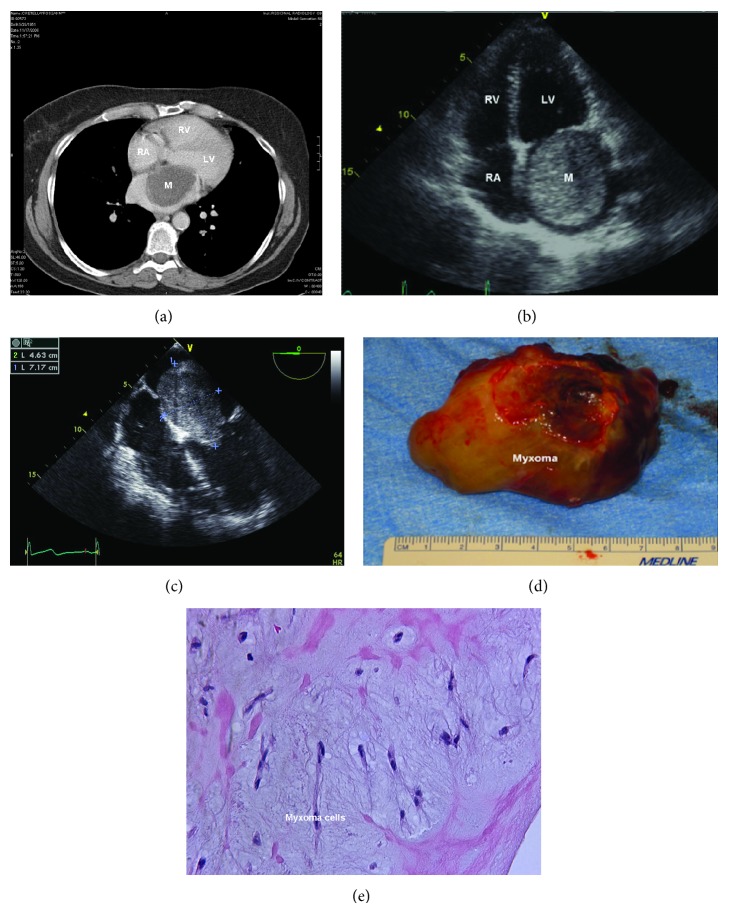
(a) Computed tomographic scan revealing a large hypodense mass (+ 30 Hounsfield units) in the left atrium. (b) Transthoracic echocardiogram (apical 4-chamber view) showing a giant, solid, smooth mass in left atrium, attached to the interatrial septum causing severe obstruction of the mitral valve in diastole. (c) Intraoperative transesophageal echocardiogram confirming the attachment of the left atrial mass (7.17 × 4.63 cm) to the interatrial septum. (d) Resected tumor (7.7 × 5.5 × 3.7 cm) having a nodular hemorrhagic surface and mucoid glistening variegated cut surface with focal hemorrhage. (e) Microscopic examination (H&E stain) showing abundant myxoid stroma and scattered polygonal cells with scanty cytoplasm typical of myxoma. Myxoma cells forming rings, cords, and nests around the capillaries with positive immunoreactivity to calretinin.

A 59-year-old woman with a longstanding history of exertional dyspnea being managed as bronchial asthma presented for evaluation of nonspecific abdominal pain. The computed tomographic scan of the abdomen revealed an incidental finding of a large, hypodense mass in the left atrium (LA) (Figure 1(a)). On interview, the patient reported frequent episodes of dizziness on leaning towards her left side. A transthoracic echocardiogram was obtained and showed a giant, solid, smooth, mobile mass in LA, attached to the interatrial septum causing severe obstruction of the mitral valve in diastole (Figure 1(b)). The patient was diagnosed with LA myxoma and scheduled for surgical resection (Figure 1(c)). The tumor was removed with its short stalk and a part of surrounding interatrial septum. A pericardial patch was used to close the septal defect. The giant resected tumor (7.7 × 5.5 × 3.7 cm) had a nodular hemorrhagic surface and mucoid glistening variegated cut surface with focal hemorrhage (Figure 1(d)). Histopathology demonstrated abundant myxoid stroma and scattered polygonal cells with scanty cytoplasm, typical of myxoma. No mitotic figures were seen. Myxoma cells formed rings, cords, and nests around the capillaries and were immunoreactive to calretinin (Figure 1(e)). Patient tolerated the procedure well and her symptoms resolved.

## 3. Discussion

Myxomas are thought to originate from multipotential mesenchymal cells of the endocardium [1]. They are benign but may have abnormal DNA content [2]. Around 7% of cases are familial, Carney syndrome (autosomal dominant multiple neoplasia and lentiginosis) being the outstanding example [3]. Myxomas are usually solitary but multiple atrial, biatrial, atrioventricular, and biventricular tumors have been reported. They usually range in size between 0.4 and 6.5 cm [4]. Recurrence rate is about 5% [5]. Symptoms could be nonspecific. Common presentations include manifestations of left ventricular failure, syncope (secondary to mitral valve obstruction), embolic, and constitutional symptoms (fever, fatigue, weight loss, and increased erythrocytic sedimentation rate). Constitutional symptoms are related to increased plasma levels of interleukin-6 [2]. Large tumors are related to atrial fibrillation. The presence of a mobile component on transesophageal echocardiogram is found in most myxomas presenting with neurologic manifestations. The tumor could be fatal secondary to a massive embolic stroke or mitral valve obstruction. Hence, prompt surgical resection is advised to avoid embolic events and sudden death [6].
